# Scientific document processing: challenges for modern learning methods

**DOI:** 10.1007/s00799-023-00352-7

**Published:** 2023-03-24

**Authors:** Abhinav Ramesh Kashyap, Yajing Yang, Min-Yen Kan

**Affiliations:** 1 ASUS Intelligent Cloud Services (AICS), Singapore, Singapore; 2 School of Computing, National University of Singapore, Computing 1, 13 Computing Drive, Singapore, 11741 Singapore

**Keywords:** Scientific document processing, Natural language processing

## Abstract

Neural network models enjoy success on language tasks related to Web documents, including news and Wikipedia articles. However, the characteristics of scientific publications pose specific challenges that have yet to be satisfactorily addressed: the discourse structure of scientific documents crucial in scholarly document processing (SDP) tasks, the interconnected nature of scientific documents, and their multimodal nature. We survey modern neural network learning methods that tackle these challenges: those that can model discourse structure and their interconnectivity and use their multimodal nature. We also highlight efforts to collect large-scale datasets and tools developed to enable effective deep learning deployment for SDP. We conclude with a discussion on upcoming trends and recommend future directions for pursuing neural natural language processing approaches for SDP.

## Introduction

A large number of scientific articles are published everyday, making it challenging for researchers to stay abreast of current developments in their fields. In biomedicine alone, researchers publish a new article every 2 min on average, resulting in more than a million publications per year [101]. This makes it difficult for researchers to find and read publications, and synthesize and summarize them. Automated ways to help them in their daily activities are necessary. Automatic scientific document processing (SDP) is such an avenue that it can enhance and simplify research tasks. For example, SDP-enabled digital libraries, such as Semantic Scholar^1^ and Aminer,^2^ equip researchers with tools that search and filter papers, track citation counts, extract figures, tables, and equations, among other functions.

**Fig. 1:**
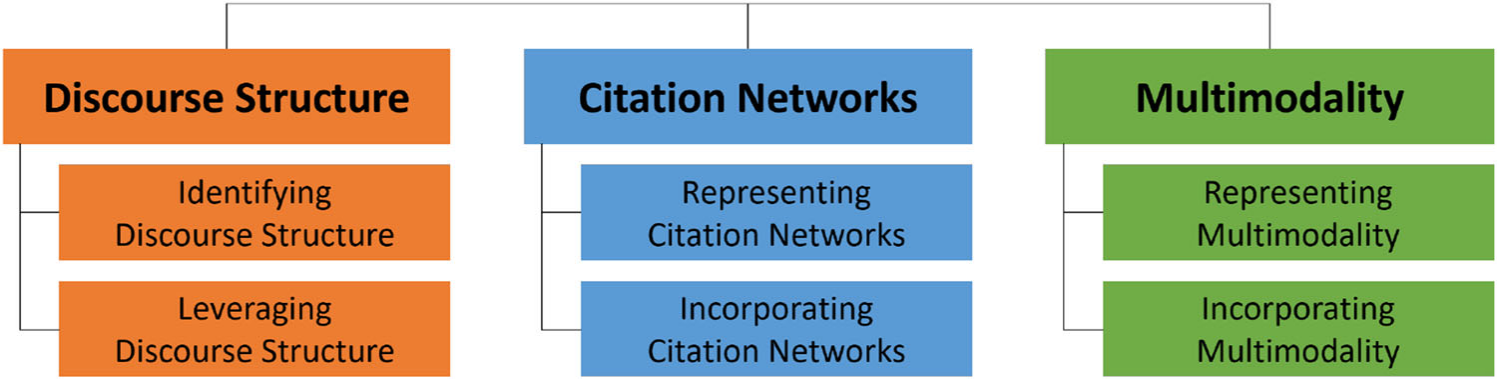
We organize this article by the challenges offered by SDP to modern machine learning methods. The first challenge is in modeling and leveraging scientific discourse structure to improve the performance of models. A second challenge is in modeling and leveraging the interconnected nature of scientific documents for neural network processing. Multimodality forms a final challenge, as neural methods currently handle non-textual modalities poorly

The current wave of modern neural network methods has enabled useful applications such as automatic summarization [16], the extraction of figures, tables and mathematical equations [31], and the recommendation of articles based on user interests [12]. It is natural to bring such advances to SDP, but so far the peculiarities of scientific publications have challenged conventional neural network models. For example, long short-term memory networks (LSTMs) [79], which are widely used, can process only a few hundred words at once, while scientific publications are much longer. This requires innovations in neural network architectures to enable the processing of longer text [14]. Similar innovations are required to consider the hierarchical discourse structure of scientific documents (Sect. 2). Given these challenges offered by scientific document processing, can researchers reinvent modern methods to handle these peculiarities?

New methods and strategies to deal with the unconventional setting present in SDP have been on the rise. This warrants a literature survey to better understand the challenges and opportunities offered by SDP and the techniques developed to address them. Here, we synthesize the different challenges posed by SDP for neural networks, concluding each challenge with a representative rhetorical question.

First, scientific documents from STEM (science, technology, engineering, and mathematics) fields follow a specific, conventionalized discourse structure [9]. In addition to identifying the different sections of the document, applications must effectively utilize this information for SDP tasks, such as summarizing and classifying citation intents, among others [32, 33]. For example, interpreting the purpose of equations is section-dependent; ones introduced in the evaluation section may explain how a work is quantitatively evaluated, but ones introduced in a method section may describe key mathematical proofs. *How can we adapt vanilla neural architectures to deal with hierarchical document structure?*

Second, scientific work has the intrinsic characteristic of referencing prior work through citations. Citations serve many purposes. Citations are used to acknowledge the existence of closely related works, to refer to background knowledge beyond the scope of the current work, and to compare or contrast with other works, among others. The interconnected nature of scientific documents requires combining information from multiple documents to solve tasks such as citation recommendation [50], paper recommendation, and summarization. However, neural networks largely consider only one sentence or paragraph at a time. *How do we adapt neural networks to effectively incorporate information from multiple related documents?*

Third, scientific work is rich in its multimodal representations. In many subject areas, scientific work incorporates tables, figures, and diagrams as embedded artifacts within the document itself. There are also auxiliary artifacts related to the work, inclusive of data, computer code, and other forms of attachments, that together with the manuscript provide a complete scientific package. *How can we represent and leverage such multimodal information to improve performance on key SDP tasks, and how do these multimodal artifacts enrich such tasks?*

We provide a review of the literature that answers these questions. Our contributions can be summarized as follows.

1. We identify three specific challenges (cf. Figure 1) that SDP poses to modern neural network learning models: discourse structure (Sect. 3), citation networks (Sect. 4), and multimodal data (Sect. 5). Then we outline the techniques to adapt such methods to overcome these challenges.
2. We collate and compare recent tools, datasets, and other resources that have been contributed by the SDP community (Sect. 6), which can serve as a starting point for parties in investigating possible solutions for SDP tasks.
3. We outline our vision for future challenges in SDP, especially considering how advances in neural network learning can be incorporated to forge meaningful progress for SDP (Sect. 7).

## Background

**Fig. 2:**
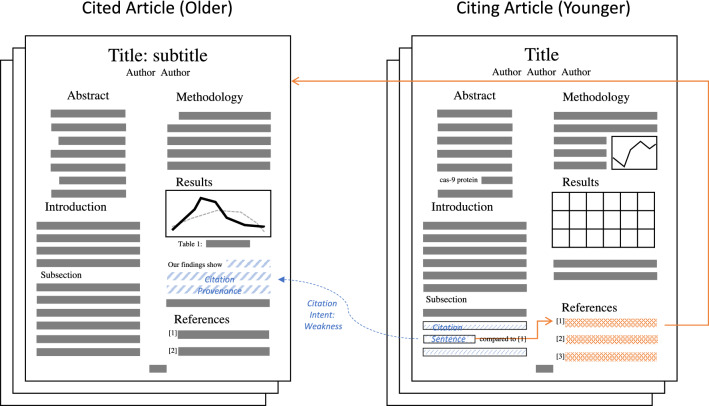
(Best viewed in color) Pictorial representation of the structure of a scientific document and its related terms. Articles typically contain an abstract and sectioned discourse such as introduction and methodology [169]. There are other discourse elements such as tables of results, figures, and sections, which play an important role in SDP. One article (*citing article*) may *cite* another article (*cited article*). The sentence which makes the citation is called the *citing sentence*. Relevant sentences before and after an anchoring *citing sentence* are called *citation text* and also play a role for SDP tasks like citation intent classification. The text relevant to a citation in the cited article is called its *provenance*. The article may contain *keyphrases* useful to indexing and searching; in the citing article, *cas-9* is a such keyphrase mentioned in the abstract

We start by defining scientific document terminology and some representative tasks. These will feature throughout this article, although the tasks are certainly not exhaustive. Figure 2 illustrates the terms.

We conclude this background section by reviewing modern machine learning models that have spurred significant advances in the underlying methods for performing SDP tasks: modern neural networks. Although their mathematical underpinnings were present decades ago, only recently were such models sufficiently expressive to accurately capture detailed patterns from large amounts of textual and visual data. A basic review of such models helps frame the challenges we have identified.

### Terminology

*Discourse structure*: Scientific articles are divided into logical parts. Documents follow a conventionalized structure and typically contain an abstract (a summary of the paper), followed by the introduction, related work, methodology, and experimental results, often in this order. They are typically identified by their *section titles* or *headers.* We consider the logical organization of scientific publications as a discourse structure that aids in the discussion of scientific material. The logical parts have their own function and style. For example, the introduction provides the broader context of the research and mostly contains text, whereas the experimental section describes the experiments and may contain figures, tables, and mathematical expressions.

*Citation*: *Citing articles* (Fig. 2, right) refers to older *cited articles* (left) using citations. Citations establish the claims made by authors, refer to methods and datasets, and credit the foundational work of other related papers. A *citation marker*—conventionally indicated with a number or an abbreviated form with the authors’ name (indicated by the text “compared to [1]” in the figure)—marks the citation.

*Citation context*: The text span around the citation contains contextual information, such as the reason for making the citation, information about the cited article. The text span (inclusive of the citation marker) may be limited just to the containing clause or sentence, but also may scope significantly beyond (both before and after) the immediate sentence. An example is marked in Fig. 2), where the context continues to the following sentence and is marked in light blue.

*Citation intent/citation function*: A citation can be made for various purposes: referring to background knowledge, comparing, contrasting with another paper, and providing evidence to corroborate a statement. The citation intent provides the qualitative purpose of the citation, in contrast to *citation count* which merely provides the frequency of the citation of an article. The citation in Fig. 2*compares* with another paper.

*Citation provenance*: An appropriate citation can refer to a specific text span in the cited article or, generally, to the entire work. The text spans in the cited article that are relevant to this citation are termed its citation provenance [162] (Fig. 2, blue text in the cited article). Occasionally, authors do erroneously cite work—i.e., the cited work does not contain evidence supporting the citation context.

*Citation string*: The bibliography or the reference section of a scientific article contains a list of references, conventionally found as footnotes or endnotes (Fig. 2, orange cross-hashed text in the citing article). Every item in the list—individually termed citation strings—contains necessary information to uniquely identify and locate the work: its authors, the publication venue and year, and other information.

### Tasks

Scientific document processing encompasses many tasks for many stakeholders. Instead of reviewing all such tasks, our purpose is to highlight the challenges posed by scientific document processing for modern methods. As such, we focus our discussion on indicative exemplars that align with our three challenge areas and the approaches that address them.

*Keyphrase extraction*: Keyphrases are words and phrases that describe important aspects of an article: its main topic, materials or reagents, or methods [73]. The abstract section of Fig. 2 shows the “cas-9 protein”—a protein associated with gene editing. Keyphrases aid different SDP tasks: indexing and searching documents by topics, clustering documents, recommendation, etc. Keyphrase extraction identifies and filters keyphrases from a publication.

*Keyphrase generation*: Keyphrases may also generalize salient topics or be selected from a controlled vocabulary (keyphrase classification) and, as such, may not actually appear in the text. Topics can be described after reading and understanding a document. Generation differs from extraction, aiming to produce pertinent keyphrases including those that do not appear as is.

*Document summarization*: Summarization condenses a long document while still preserving key information. For scientific text, the summaries should contain background information, results given the context of related papers, the document’s contributions, and their implications. Such salient information may appear in different parts of the scientific document. *Extractive* summarization extracts important sentences as is from the document, while *abstractive* summarization does not draw sentences verbatim from the source. These forms of summarization are common to standard text corpora, such as news articles. However, the SDP summarization can capitalize on its unique structure of citing and cited papers. Citing papers provide the community’s perspective of the paper and can be considered a complement to the abstract. Summarization that considers citations is called citation-based summarization.

*Citation and paper recommendation*: Researchers may use aids to find relevant publications to cite or to read. For example, given a sentence “adversarial autoencoders generate realistic images and show improved performance,” Makhzani et al. [123] is an appropriate citation providing background information about adversarial autoencoders. Citation recommendation aims to suggest appropriate citations considering the statement, the context around the statement, the aim of the publication, and the coverage of citations in prior sections.

*Citation intent classification*: Citations are not equivalent. Citations express different sentiment—positive, negative, no sentiment—about the cited article [216]. They may also reflect different purposes: referring to background knowledge and indicating weaknesses, similarities, differences, or improvements with respect to other publications. This task’s aim is to automatically identify such intents. The results can then be used to selectively read specific related literature (e.g., list papers that provide an improvement over a target paper or ones that create a benchmark evaluation metric).

### Neural networks

Supervised machine learning algorithms train a model using a set of examples—also termed as labeled data points. To predict outcomes, traditionally, features are extracted from raw data. However, in real-world applications, manually extracting features is difficult. Modern approaches solve this by automatically learning such features. Modern neural network approaches specialize in learning such complex features by means of using multiple layers that forms the network architecture. This form of learning in which the user specifies the text and its corresponding label—without engineering any features—is called *end-to-end learning*. It is this ease of use, coupled with its impressive performance gains, that has led to the rapid adoption of modern neural methods in many communities.

**Fig. 3:**
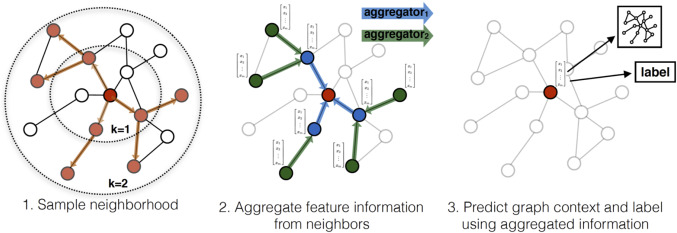
An example of a graph neural network. It starts with sampling a set of neighborhood of the node. Within each layer of the graph, the NN-based aggregator aggregates information from a node’s neighbors. The final representation of the node is used to predict graph context and label [71]

*Recurrent neural networks (RNN)*: Standard neural networks process inputs from beginning to end in one pass: They pass information to subsequent layers but do not reuse intermediate information, although such intermediate states can be useful. The recurrent architecture addresses this by reusing these intermediate results from previous data points for future ones. RNNs are used to process sequences, where information from previous states plays an important role for the current state, such as text where previous words have predictive power in determining the next words as well as the overall meaning. In SDP, they are commonly used to process sequences of text from scientific articles for tasks like classification. While RNNs made significant progress in text processing, transformer models [44] have been prominent in the recent past. We refer the reader to an online post^3^ for an intuitive introduction.

*Convolutional neural networks (CNN)*: Another useful variation of general neural networks are convolutional neural networks. CNNs specialize in understanding spatial inputs like images, 2D blocks of text, tables, or figures. They build representations by considering spatially local information, like a patch of image, and further compose these local representations to build representations of larger spatial blocks or volumes, until the entire input is considered. In SDP, CNNs find their application for tasks such as identifying discourse structure, where considering the entire article at different resolutions is critical. Other applications like automatic understanding of tables and figures also use CNNs, as these inputs also have locally spatial regularities in the form of recursive, decomposable, top-down structure.

*Graph neural networks (GNN)*: Unlike images or text, a graph consists of unordered nodes of no fixed form. Graph neural networks handle the complex topology of a graph by adopting the idea of convolution to look at only the local network neighborhoods of a node. Convolution builds representations using information from its local neighbors, as in CNNs; but in GNNs, the set of spatially local neighbors is dictated by the random graph and not fixed as in 2D images and tables. As shown in Fig. 3, GNNs determine a computation graph for each node and learn each node’s representation by aggregating its neighboring information. The resulting representation comprises both the node features and the graph topology. GNNs produce state-of-the-art performance in node classification, link prediction, and clustering tasks.

RNNs, CNNs and GNNs are all basic variants that specialize in handling regularities in the input, specifically, sequential, fixed spatial, and random spatial regularities, respectively. All of these require sufficient data to set the weights of these models appropriately. We end by mentioning pretraining as a means to obtain good initial weights for many of these models.

*Pretrained language models*: Supervised learning requires large quantities of labeled data, often difficult to obtain. Can we first learn general information from the unlabeled, publicly available text on the Web? Can we further use this information to learn appropriate tasks with limited labeled data? The answer to these two questions is “yes,” where the first task is referred to as “pretraining,” and the second as “fine-tuning.” This pretraining revolution first uses unlabeled text to learn useful associative patterns, which address many shortcomings in language understanding and meaning interpretation. While pretraining in natural language research was first limited to the word level (Word2Vec), computer vision researchers trained deep CNNs in the ImageNet dataset for image recognition [42], learning generic, hierarchical representations of images such as edges, curves, and textures. They further fine-tuned these representations for advanced computer vision tasks such as object detection [154] and image captioning [89].

This pretrain-and-fine-tune task decomposition is applicable for text processing as well. Researchers trained deep neural networks to predict how likely the next word is given a piece of text (language modeling). The aim is to learn general patterns of language while performing this task. As of current, the most extensively used pretrained language model is BERT [44]. After learning the general representation, fine-tuning the language model improved performance of other NLP tasks: sentiment analysis, semantic text similarity, assessing the linguistic acceptability of text, etc. [178]; all of such fine-tuning operations have been applied successfully to BERT.

## Discourse structure: challenge #1

Scientific discourse structure divides the document into logical parts. We consider any such logical organization as *discourse structure*. Leveraging such a structure is crucial for SDP tasks. As an example, consider keyphrase extraction. Keyphrases are often found in particular sections (e.g., title, abstract, and introduction). However, neural networks do not natively incorporate discourse structure. Such a sequential, end-to-end processing largely disregards the hierarchical discourse structure of the scientific publications. Even the first step in identifying the structure of the discourse is challenging. We discuss methods to identify discourse sections (Sect. 3.1) and then methods to take advantage of them (Sect. 3.2) to improve the performance of downstream SDP tasks.

**Table 1:** Overview of different neural network-based prior works that identify the discourse structure of scientific documents

Paper	Aim	Approach	Code availability
Soto and Yoo [161]	Identify title, authors, abstract, body, etc ***(Global)***	Faster-RCNN (***Vision***)	
Science Parse	Identify title, authors, section headers, etc. ***(Global)***	Bi-LSTM (***Text***)	
Yang et al. [199]	Identify title, authors, abstract body, etc. ***(Global)***	CNN + text embeddings (***Hybrid***)	
Dasigi et al. [41]	Identify the problem, method, implications, etc. within the experiment section ***(Local)***	LSTM (***Text***)	$\checkmark$
Jin and Szolovits [85]	Analyze the abstract of the scientific paper ***(Local)***	Hierarchical LSTMs (***Text***)	$\checkmark$
Madisetty et al. [122]	Parse mathematical equations ***(Global)***	Bi-LSTM (***Hybrid***)	
Wang and Liu [184]	Generate mathematical latex equations ***(Global)***	LSTM + CNN (***Hybrid***)	
Siegel et al. [160]	Extracting figures ***(Global)***	CNN (***Vision***)	$\checkmark$
Banerjee et al. [13]	Identify background, technique and observation from abstracts ***local***	Bi-LSTM ***Text***	$\checkmark$

**Aim** captures the diversity in the end goal of articles. The **Approach** identifies the neural network method used to identify discourse structure. **Code Availability** refers to availability of the project code; where available, URLs to codebases are listed in the respective reference in the bibliography

### Identifying discourse structure

The backbone of several SDP tasks requires the identification of logical sections in a scientific document, which is a necessary step to leverage discourse structure in downstream tasks. Here, we review neural network methods that analyze scientific publications, although there has been significant informed work on other structured documents such as receipts [43, 90, 91], web documents [135], business documents [185], examination papers [120], among others. The reader is invited to refer to [49, 166] for a comprehensive survey on other types of documents. Table 1 shows the comparison between neural methods to identify the structure of scientific discourse. We inventory such NN-based methods based on two dimensions of comparison: the discourse elements identified (Aim), and the NN approach used (Approach).

1. *Aim*: Kan et al. [88], a precursor non-NN method, associate every line with one of 23 line functions (i.e., section header, title, page number, figure caption, etc.) and further classifies sections (i.e., a section header accompanied by body text) into one of 13 generic logical types (introduction, related work, methods, etc.). Recent NN methods [199] have greatly improved the extraction of similar discourse structures, albeit only at the coarse-grained section level and without logical types (e.g., Allen AI’s Science Parse; cf. Table 3). In addition to text, scientific documents incorporate visually distinct figures, tables, and mathematical equations. They provide a summary of the methods and results which aid in finding related papers, among other uses. NN models also extract such multimodal objects, such as figures [160] and tables [161]. We limit our discussion to the discourse structure of text, leaving the details about the multimodal elements to Sect. 5. All the papers discussed till now consider a high-level structure within the document—such as the abstract, introduction, and methodology—which we term *global*. Identifying global structure aids in document-level tasks such as recommendation, summarization, and retrieval. Finer, *local structure* within individual sections can help researchers assess the appropriate reading and writing strategies from other works. For example, Jin and Szolovits [85] identify the background, objectives, methods, results, conclusions, and order within the abstract section of scientific publications. They analyze the prevalent order in scientific publications, which can help researchers structure their own abstracts. Banerjee et al. [13] also identify the local structure of abstracts. They first train a model on biomedical data to identify categories before fine-tuning on a small set of computer science articles. Similarly, Dasigi et al. [41] identify the different components of the experiment section: the problem, the goal, and the results of the experiments. Analyzing fine-grained sections aids in automatic literature review by grouping works that use similar methods and outcomes.
2. *Approach*: Researchers use two types of neural network to identify discourse structure: convolutional neural networks (CNN) and recurrent neural networks (RNN) (cf. Section 2). Researchers choose CNNs or RNNs depending on the way they treat scientific publications: preferring RNNs when they treat them only as textual sequences [41, 85].

*Recommendations*: Neural methods are increasingly used to identify the discourse structure of the textual content. We find that the community is focused on identifying the global structure of scientific publications. But analyzing the local structure of publications can result in tools for efficient reading and writing for scholars [13].

**Table 2:** Overview of methods incorporating discourse structure for an end task in scientific document processing

Paper	Approach	Task	Method description	Code availability
Cohan et al. [33]	End-to-end	Abstractive summary	Hierarchical Attention Networks	$\checkmark$
Xiao and Carenini [188]	End-to-end	Extractive summary	Attention over word, sentence and document representations	$\checkmark$
Collins et al. [35]	End-to-end	Extractive summary	Categorical feature added to neural network	$\checkmark$
Gidiotis and Tsoumakas [61]	Divide-and-conquer	Abstractive summary	Pointer generator RNNs per section	
Chaturvedi et al. [22]	Divide-and-conquer	Extract + abstractive summary	BioBERT + Graph-based extractive summaries + BART	$\checkmark$
Kobayashi et al. [99]	Divide-and-conquer	Citation recommendation	Word embeddings + Simple classifier + Graph neural networks + Recommendation	
Chen et al. [30]	End-to-end	Keyphrase generation	Attention-based encoder–decoder RNNs	
Cohan et al. [32]	Multitask	Citation intent classification	LSTM networks	$\checkmark$
Su et al. [163]	Multitask	Citation provenance and intent	LSTM networks	$\checkmark$
Cachola et al. [16]	Multitask	Abstractive summary	BART	$\checkmark$
Ye and Wang [203]	Multitask	Keyphrase generation	Encoder–decoder RNNs	

**Approach**: We identify three main approaches to incorporate discourse structure into scientific articles. *End-to-end, Divide-and-conquer, Multitask* learning. **Task**: Incorporating discourse structure benefits different SDP tasks, and we identify the different tasks tackled by different works. **Method Description**: A brief description of the neural networks used in the work. **Code Availability**: where available, a hyperlink to the codebase is given in paper’s reference

### Leveraging discourse structure

Modeling the discourse structure of scientific publications provides several advantages to downstream SDP tasks: division of long documents into small logical sections, providing prior information for certain tasks (citation intent classification), and allowing the comparison of publications based on sections. Here, we review NN methods that use discourse structure to benefit SDP tasks. Table 2 summarizes works based on their task, the modeling approach used to incorporate the discourse structure, and the NN architecture used.

1. *Task*: The modeling of discourse structure informs the downstream models in performing the key characteristic SDP tasks (i.e., document summarization, keyphrase extraction, and citation intent classification). Summarization approaches based on models of standard text [156, 157] underperform on scientific documents, due to their long and conventionalized document structure. Knowledge of discourse structure pinpoints where specific forms of knowledge lie (key aspect of the methodology in *Methods*, discoveries in the *Results* section), allowing summarization methods to model different functional aspects of the document. Both extractive [35, 188] and abstractive SDP summarization [16, 22, 33, 61] leverage discourse structure for this reason. In addition to summarization, keyphrase extraction benefits from incorporating discourse structure. As keyphrases capture salient information about the paper, they concentrate within certain sections, such as in the methodology or the introduction. Similarly, other works [30, 100, 203] observe that the title of the document largely overlaps with keyphrases, and that modeling titles for keyphrase extraction improves performance. The intent of the citations also depend on the section in which they appear. For example, most computer science papers compare and contrast with other works in the literature review section, while the citations in an introduction provide background knowledge. Therefore, it is important to incorporate discourse information for automatic classification of citation intent. [32, 164] are exemplars that incorporate the discourse structure in neural networks for citation intent classification.
2. *Approach:* We identify three ways that neural networks incorporate discourse structure: *end-to-end, divide-and-conquer,* and *multitask*. *End-to-end*: End-to-end methods build continuous representations for a discourse section, starting from sequential text. Since forming representations for longer scientific documents is harder for neural networks, they compose representations of smaller elements like words [130, 146] and sentences [36, 98] to form representations of larger elements such as whole documents. Hierarchical attention networks [200] are one such framework that combine continuous word representations with sentence representations, further combining them to obtain section- or document-level representations using the attention mechanism [11]. Building document- and section-level representations using hierarchical networks has been shown to be useful for abstractive summarization [33], extractive summarization [188] and keyphrase generation [30]. Incorporating discourse structure to solve a SDP task using the popular end-to-end paradigm requires complex neural network architectural changes. Since scholarly documents are long, using neural networks to sequentially process documents is inefficient and ineffective, due to high computation and memory requirements. *Divide-and-conquer*: To ease the burden of pure end-to-end learning, divide-and-conquer approaches are helpful. As the discourse structure of the documents naturally helps to divide the problem into smaller ones, smaller section-wise solutions can be solved and combined later. For example, a separate summary can be formed for different discourse sections and combined to form a final summary. Such an approach is popular for summarizing [22, 61], citation recommendation [99], and paper recommendation [143]. *Multitask*: Multitask learning considers a main task and a complementary auxiliary task together. The two tasks exploit the commonality and the differences between them to improve generalization [19]. It has been used in innovative ways to employ discourse structure in neural networks for various SDP tasks. Generally, an objective—related to the discourse section to be incorporated—is added as an auxiliary task to the main SDP task. For example, Cohan et al. [32] predict the title of the section as an auxiliary task for citation intent classification. Su et al. [163] show that the detection of citation intent and citation provenance can enhance each other’s performance in a multitask setting. Cachola et al. [16] use multitask learning to generate extreme summaries of scientific documents that are a couple of sentences long; perfect as search result snippets. They use an auxiliary task of title generation, finding this multitask setup improved performance. Similarly, title generation has been used as an auxiliary task for keyphrase generation [203]. Multitask learning has multiple advantages: improving the generalization ability of a model’s solution, and efficiently modeling a new task with minimal data. This is accomplished by incorporating information from discourse elements related to the task to obtain performance gains. *Commonalities and differences*: Most end-to-end-based approaches compose word and sentence representations to form discourse-level representations using attention-based RNN networks [11]. Cohan et al. [33] uses RNN encoders to build sentence-level representations from word representations [33, 188]. Once the document has been encoded, such methods employ attention in the decoder to model differing levels of section importance when generating summaries. Similarly, Xiao and Carenini [189] uses the intuition that a decision to include a sentence in the extractive summary depends on not only the importance of the sentence within a section, but also on its importance within the entire document. To achieve this, they use an attention mechanism over the hierarchically composed word, sentence, discourse section, and document representations to decide whether to include the sentence in the extractive summary. On the other hand, instead of making complex architectural changes that become infeasible to handle long documents, the *divide-and-conquer* approaches use a pipeline-based approach. For example, Gidiotis and Tsoumakas [61] first classify an annotated summary sentence into different discourse sections, creating a pseudo-section–summary supervised pair. Then they use a neural network for abstractive summarization of every section before combining them. The *divide-and-conquer* also has inspired a hybrid *extract–abstract* summarization approach that first extracts a sentence and then perform abstractive summarization. This method has become popular not only in non-scientific domains [105, 129, 190] but also in scientific documents [22]. *Divide-and-conquer* approach can combine multiple existing methods to achieve a better result [22, 61, 99]—allowing easy switch of components for more advanced ones, it handles longer documents effectively without complex changes to neural network architectures. Multitask-based approaches share a few layers of a neural network like LSTMs [32, 163] and then use separate layers for each complementary task to capture task specificity. Although earlier works used multitask learning for mainly classification tasks [32, 163], it has found a resurgence in text generation [16, 203].

*Recommendations*: Incorporating discourse structure into neural network modeling is essential to improve SDP tasks. Although traditional methods can incorporate discourse structure by considering them as additional features, neural network methods best model discourse structure by making appropriate architectural changes to build hierarchical representations to incorporate such information. End-to-end methods for incorporating discourse representations are currently limited by the length of text that can be processed at once—ineffective in handling long paragraphs or documents. New methods such as [14] have attempted to alleviate this challenge recently. Our opinion is that the *divide-and-conquer* approach can leverage existing technologies creatively and provide more practical solutions.

## Citation networks: challenge #2

**Fig. 4:**
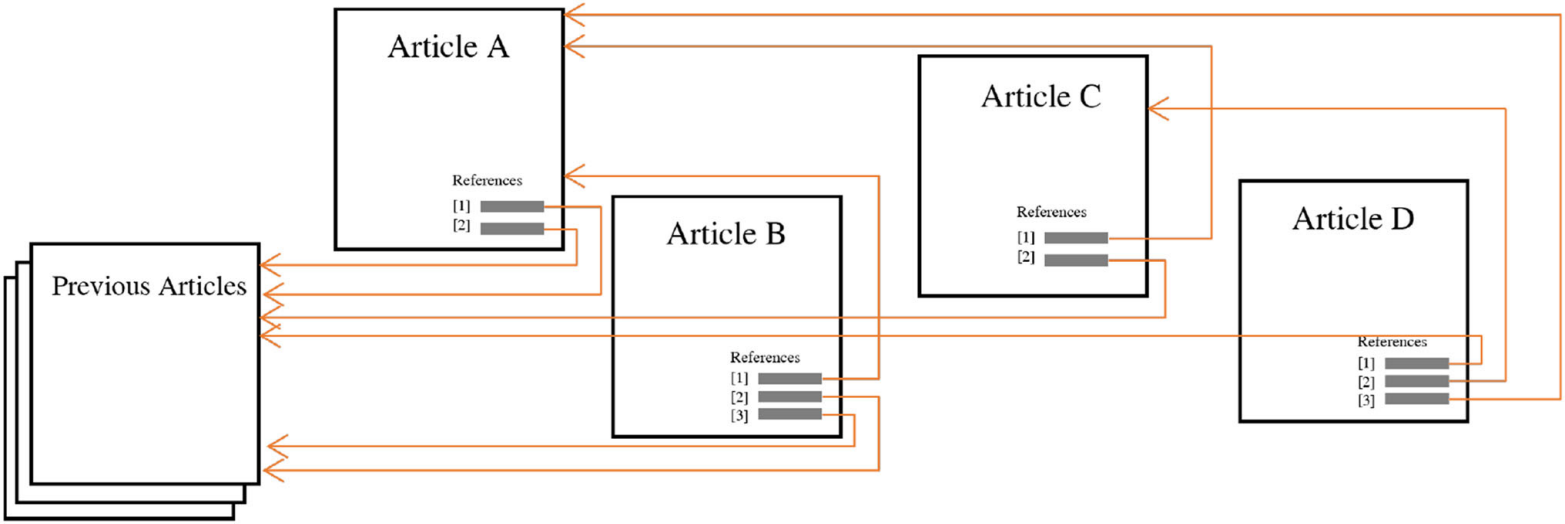
Pictorial representation of a citation network. Articles cite the previous articles and are cited by future articles. The number of citation consisted of and received varies across different articles

Scientific documents link to each other using citations (Fig. 4). Citations relate the scholarly work to the background and context of the works and relevant concepts. A collection of papers forms a citation network or graph, where edges model the citation relationship between two papers. The bibliographic details (i.e., author, year, publication venue, title) and the content of a paper are the node features in such a citation network. The citation context highlights the purpose of a citation or the edge type of citation network edge.

To properly utilize citation information under a neural scenario, it is essential to generate effective numerical representations for both the node and edge features of a citation network. In addition to component representations, an appropriate neural architecture needs to be chosen to represent such citation networks as a whole. Despite the success of CNNs and RNNs in handling computer vision and natural language processing tasks, they are designed to handle tabular (raster) or sequential data. However, citation networks have neither spatial logic nor fixed ordering of nodes. Graphical representation models built with NNs, such as graph convolutional networks, yield superior performance in extracting the node features and graph structure automatically. We start by introducing the application of the NN-based graph representation methods on citation networks and then describe how these representations are utilized in SDP tasks.

### Representing citation networks

Although NN-based methods achieve outstanding graph representation performance, naïve application of such methods on citation networks fall short in performing well. While some works have explored how to handle the challenges posed by the unique characteristics of citation networks, many areas remain to be explored.

*Node characteristics*. Bibliographical information includes nominal data (e.g., author, publication venue, and year) as well as textual strings (e.g., title, abstract). We discuss examples within these two classes of data in turn.

Author information can be utilized to assist in topic modeling, as authors preferentially publish on only certain topics. However, when a large number of co-authors are present on a publication, it becomes more difficult to characterize the work, making a low-dimensional representation of author essential. A common method is to aggregate all the titles of an author’s publications [205]. Sugiyama and Kan [167] extend this representation method to the referenced paper and papers citing the scholar’s work, while Bulut et al. [15] include the documents related to the scholar’s research field. Ebesu and Fang [47] build two networks to learn the embedding of cited and citing authors separately, and incorporate the output during decoding. Holm et al. [80] model the author and venue with the total citations received.

In our view, the utilization of the venue and year information is still superficial in contrast to the many works that utilize author information. A venue representation aggregates all work grouped under the same research cluster could help to create a better representation for the target paper. Further, the prestige of a publication venue (e.g., impact factor of a journal or ranking of a conference) is important in deciding the influence of the paper. The publication year of a work can be used in tasks like citation recommendation, to account for its fading novelty as a publication ages [127].

Most works extract textual features from title and abstract using a recurrent network (specifically, LSTM) [180, 201] or pretrained language model [84] for graph representation learning. While titles and abstracts provide summaries of documents, the full text contains information not available in these two. However, the structured and lengthy nature of scientific documents poses challenges for standard RNN or transformer architectures to encode. Future works should also consider a faceted representation as introduced in Sect. 3, based on the needs of downstream SDPs.

*Edge characteristics*. The edge in a citation network represents the citation relation between two papers. Such edges are directed from the citing paper to the cited one and can be associated with a time lag and its citation context. Li et al. [110] build graph neural networks (GNN) and handle the directions by using only the incoming nodes in identifying important information during aggregation. Jin and colleagues [86] model the citation directions using two separate networks. The time lags are the time difference between publication date of the cited paper and citing paper, which shows the diffusion speed of information from the cited paper. Fu et al. [56] model the time lags using Monte Carlo simulation. The resulting citation network is then deployed to improve the results of a citation recommendation task.

The citation function, or the reason a citation is included, can be extracted from citation context [173]. The citation context often implies the function and polarity (negative/neutral/positive) of a citation too [2, 60]. Future works will be able to produce better representation of citation relation by incorporating these derived features. Citation context is also the basis for certain SDP tasks. For example, a context-aware citation recommender aims to find papers that best match a given citation context [40, 84, 171, 180, 197, 198, 205]. And a stream of summarization models use citation context as the input for summary construction [111, 201, 208]. Citation context can also be used to generate document embedding by treating the citation context as sentence and citation as word [58, 72].

In summary, a node in a citation network is associated with rich information including metadata (author, year, publication venue, title, abstract) and a long document content. The features of a citation include date, function, and citation direction. Although research has been done to utilize these features, the incorporation of publication venue and year, the textual representation of the document content and its application of citation function/intention derived from citation context remain as challenges in building an effective and universal citation network representations.

### Incorporating citation networks

Appropriate modeling of citation networks using the above methodologies significantly boosts performance on various SDP tasks. Although many applications such as citation forecasting [74] and extracting emerging concepts from scientific documents [97], we select two key SDP tasks—citation recommendation and citation-based summarization—where network representation is critical and forms the basis for their approach.

*Citation recommendation*. Citation recommendation (and also Sect. 2.2) is accomplished based on either the global or local context. A global citation recommendation model recommends papers according to their similarity with a given document. Gupta and Varma [68] randomly generate a set of short walks from the citation network with each node as the origin, and find the node representations to maximize the probability of the generated walks. This topology representation is combined with semantic features to yield document representations by maximizing the correlation between the two. Another method based on random walks generation proposed by Guo et al. [67] merge the citation relation and content similarity between papers as the input for node representation learning. Other works treat all bibliography entities as nodes and build bibliographic networks that contain nodes corresponding to different entities such as author, venue, and paper (heterogeneous bibliographic networks or HBNs). Cai et al. [17] represent an HBN with an adjacency matrix to train a GNN-based recommender. Models proposed by Ma and colleagues [118, 119] generate node embedding by extracting topological features from HBNs using different meta-path proximity methods. Mu et al. [133] model an HBN as a multilayered graph (for authors, papers and words, respectively), while incorporating user queries into the graph to yield a query-focused recommender.

In contrast, local (or context-aware) citation recommendation produces recommendations for a specified text span where a citation is needed. Such applications can benefit writing assistance and can incorporate citation impact prediction as well [139]. These recommenders generate a representation for both the citation context and each candidate paper, and make recommendation based on the similarity score between the two. Jeong et al. [84] predict the probability of recommending a paper based on the concatenation of citation context embedding encoding generated using BERT, and a document embedding produced with GNN. Medić and Snajder [126] also tackle the local citation recommendation problem, incorporating article title and abstract information—hinting at the productivity of incorporating discourse structural information (c.f. Section 3).

Other works compare the paper content and the given citation context directly. Neural NLP methods are commonly applied to create the text representation [40, 142, 171, 180, 197, 198, 205]. In contrast to the aforementioned works, which generate one representation for each paper, [51, 72] build two vectors per paper: one for each case of citing and being cited, respectively.

*Citation-based summarization*. Citation graphs enable improved contextual modeling of a paper and hence can improve summarization performance. As an example, certain scientific concepts may not be explicitly explained, where the document authors refer the reader to other documents through citations to explain the necessary concepts. The citation graph-based summarization model proposed by [7] incorporate this information in the referenced paper using GAT. The input features of the nodes are the textual features extracted from the abstracts of the referenced paper, inferred from an LSTM. The node representations are then fed to a LSTM-based decoder to yield attention weights, which are then applied to choose content for document summaries.

Citation sentences are usually in the form of a summary of the aim, method, or results of the cited work. A set of such sentences for the cited documents become useful data sources for generating a summary. Qazvinian and Radev [149] proposes citation summary networks where each node represents a citation sentence referencing the document of interest, and where similarity scores between nodes determine edge weights. Sentences extracted from a clustering of the weighted graph form the summary. Chen and Zhuge [26] design a multidocument summarizer employing citation sentences. They cluster the citation sentences to identify common facts and them as weights (in the same guise as attention weights) to select sentences from input documents to form a summary.

However, such citation sentences are written from the referrer’s perspective, hence tend to be subjective. Recent works solve this issue by also utilizing citation provenance. Here, citation provenance refers to the most similar to the citation sentences found in the target document, which serve as the proxy for the provenance or reason for the citation. Classifiers built with pretrained BERT [208] or CNN [111] find citation provenance by treating it as a sentence pair classification problem. Yasunaga et al. [201] use the abstract and the citation provenance as input to build a GNN. The node representation output from GNNs serves as a salience estimation in selecting summary sentences.

*Recommendations*: Incorporating citation information boosts the results of many SDP tasks. As discussed, a node representation of a document in the citation network provides a community view of the document; hence, it is useful in identifying related papers for citation recommendation. The citation context serves as a query to indicate the users’ interests in citation recommendation. As for summarization, appropriate modeling of citation networks helps in understanding scientific concepts. Citation contexts are used directly as candidate summary sentences, or indirectly as the references for document sentence extraction.

In this review, we have only concentrated on core tasks in SDP in which citation network modeling is essential. Our opinion finds that citation modeling can assist in tasks where the contextual positioning of how a publication fits in the community’s understanding is needed. Such applications extend beyond summarization and recommendation and could include trend analysis, survey paper construction, field and topic characterization, expert finding, author and institution reputation tracking, among many other future creative uses. However, the current studies apply task-agnostic graph representation, resulting in suboptimal performance. Further enhancements should be made by incorporating or attending to document information for the task of representing the citation networks. Besides, while each discipline demands its own metadata in the references [46], identifying the optimal set of metadata entities in effective citation networks construction requires further study.

## Multimodality: challenge #3

Scholarly documents are richly formatted documents, in the sense that they are not exclusively corpora of running text. Tables, figures, line art, and workflow diagrams are among some of the artifacts that are used to communicate scholarly work. Such artifacts are often embedded within the document itself (we term these *internal* artifacts) that allow a reader to peruse data to validate the findings highlighted by the authors. These internal artifacts are often accompanied by a textual *caption*, allowing these independent artifacts to be better understood and contextualized within the parent scholarly work. At the one end of the spectrum of multimodality, scholarly documents also have other *inlined* textual artifacts, usually symbolic notation—such as ones for music, chemistry, and mathematics—or domain-specific entities that are used to encode and transmit domain-specific information. At the other end of the spectrum, datasets, program code, posters, presentation slides, and videos are satellite, complementary *external* artifacts that serve to complete a scholarly work, often facilitating secondary uses of the work, inclusive of replication, communication and use.

However, many tasks requiring modality considerations appeal to methods that concern just a single modality. As such, specific, single-modality methods are utilized. For example, techniques in computer vision utilizing deep learned CNNs are used for representing and modeling internal artifacts such as figures and tables.

### Representing multimodality

To focus on the challenges in *multi*modality, we constrain our discussion to the representation of more than one modality. We summarize multimodal representation methods in different combinations of modalities, constraining our discussion to modalities common in scientific documents. As the textual modality is central in scientific document processing, we examine multimodal representations in which text is combined with other modalities.

*Text* + *Figures*/*Tables*. Figures and tables are internal artifacts commonly seen in a scholarly document, containing information not covered by the text. To generate a joint representation of texts and figures of a document, a general framework is to extract the unimodal high-level features separately and then combine the two sets of features. Anastasopoulos et al. [8] apply concatenation to fuse the textual and visual features extracted using two pretraining models. The models proposed by Lu et al. [117] and Tan and Bansal [170] are of similar structure, but fuse features differently. They consist of two single-modal networks to separately encode input text sequences and images, and a cross-modal transformer to combine modalities. Zhang et al. [209] project features from one modality to the other as cross-modal attention, and design a cross-modal transformer that models both self-attention and cross-modal attention. Li et al. [113] and Yu et al. [206] include the object tags, attributes, and relations extracted from the source image as input to ease the text–image alignment. Although they have only tested on neural images, similar methods can be applied to scientific document representation by extracting features of scientific charts, such as the data’s trend (rise and fall), minimum and maximum.

Bilinear pooling, which originated in the computer vision community, is another common method for combining features of different modalities [172]. It combines every pair of multimodal input channels. However, while powerful, this results in an explosion in feature dimensionality and can result in overfitting and poor performance. To address this, multimodal low-rank bilinear pooling [57, 96] targets to solve this dimensionality problem by means of a low-dimensional approximation. Furthermore, multiple attempts have been made to integrate the attention mechanism with multimodal bilinear pooling to improve representation effectiveness. For example, Kim et al. [95] introduces bilinear attention networks to find bilinear attention distributions of a bimodal input before proceeding with low-rank bilinear pooling.

Tables contain text content as cells, thus can be linearized and concatenated with the textual inputs [191] for multimodal representation. Although such a method requires no feature fusion, it neglects the 2D structure of tables. Table parsers [78] solve this problem by including the cell locations in the modeling process, but how to fuse the extracted table features and textual features effectively becomes a challenge.

*Text* + *Layouts*. Layout is an important visual component of a scientific document. As the relative positions of document components (text blocks/figures/tables) significantly contribute to the document’s semantics, incorporating layout information improves the multimodal representation of a visually rich document. Layout information is included as a 2D position of a document component to build its representation. For example, Xu et al. [194] build a layout-aware transformer with text tokens and their 2D positions as input. To utilize the visual information, Li et al. [112] slice the document images into rows, representing text and images with their textual/visual features and positional encodings, and then combine the two modalities using a cross-modality encoder. Wu et al. [187] divide the documents into blocks to accommodate the various size of document components, and represent the location of the block with its top left and bottom right coordinates. They design a two-level structure to encode each block, then aggregate block-level representations, and pretrain using hierarchical objectives. For scientific documents that contains multiple pages, Pramanik et al. [147] utilize a special transformer [14] network architecture, in which the attention mechanism scales linearly with the sequence length, to encode the multimodal information. The model uses page images and page numbers as inputs, in addition to token features. The system proposed by Huang et al. [83] is an extension to [194], with an emphasis on alignment between word and image patches. It projects the patches and word tokens linearly to generate contextualized vector representations. The model is pretrained to learn cross-modal alignment between words and patches.

*Recommendations*: The commonality in such representation works is that both image and visual input content can be represented by extracting their features first separately and then to apply some fusion means them to generate a joint representation. However, because of the nature of the images (natural images vs. scientific figures) and length of the document (short paragraphs vs. long documents), practitioners should make modeling adjustments based on the characteristics of the target scientific documents. Possible adjustments include replacing the generic CNN image encoder with encoders specifically designed for scientific figures [23–25], and using transformers designed to represent long documents [147]. Representing the table with a pretrained table parser has yet to be tested in representing the tabular and textual content of a scientific document. While including layout information in the modeling process improves the representation results, additionally incorporating discourse structure (Sect. 3.2) to better represent multimodal documents remains a fertile area to be explored, in our view. Similarly, the scope of what constitutes multimodality—in terms of both inlined and external multimodal artifacts—can be expanded to more holistically represent scientific discourse for downstream tasks that may benefit from this auxiliary pathways for computing relevance and similarities.

### Incorporating multimodality

Multimodal SDP tasks can operate either in a trans-modality setting (where information in single input modality is transferred or translated into another) or in a true multimodal setting (where multiple modalities are represented as input). We examine two representative multimodal SDP tasks—captioning and summarization—along these lines and conclude with our perspective of the logical future directions in multimodal SDP.

*Captioning.* Internal artifacts such as figures and tables include text captions to emphasize aspects of the data [94, 102]. For visual information contained in charts, captioning improves the recall and comprehension of the data by drawing attention to some aspect of the underlying information [75]. Due to the importance of captions in understanding scholarly document content and the presence of title-only captions, automated caption generators can assist in paper writing. Generating captions is largely a trans-modality task, with the task of appropriate encoding of the figure or table to decode an appropriate output textual caption.

*Trans-modality figure captioning*. To automatically generate captions for figures, a common process first parses the figure with a CNN decoder, and then generates caption text based on the figure content using an RNN decoder [23–25]. Attending to the labels during decoding enables the model to utilize the text contained in the figures [23]. The sequence-level training with reinforcement learning improves the generation of long captions by directly optimizing over the evaluation metrics [25]. Such end-to-end models rely largely on the quality and quantity of the training data to automatically learn the caption generation process. To improve the understanding of the caption generation problem, Qian et al. [150]’s corpus-based study collected and analyzed human-written captions, finding that a caption often consists of a set of *caption units*, which refer to an enumerated set of clause types (e.g., number and labels of items, pair-wise comparisons) describing specific types of information in the figure. As such, to automatically generate figure captions, a system should first generate caption units according to the figures, then stitch the units together using diverse patterns to form captions. Specifically, Qian et al. [151]’s later model attends to both the visual information and the metadata information of the input figure to generate accurate caption units.

*Trans-modality table captioning*. To generate table captions, in contrast, prior work first serializes table data, then processes them with a sequence encoder using pretrained language model [132]. Such models are fine-tuned for numerical reasoning, but currently are still unable to generate high-fidelity text. Therefore, Suadaa et al. [165] introduce the use of the copy mechanism during fine-tuning to reduce the occurrence of generated phrases that are irrelevant to the table.

*Multimodal figure and table captioning*. Recent work on captioning in the SDP context consider text beyond the input figure and table. This form of the task upgrades the task to a true multimodal task, as the input includes relevant body text as well. Besides being based on the target figure or table, Yang et al. [196] consider texts relevant to the figure for caption generation. The proposed system comprises of a figure parsing module and a module to identify the sentences in the body text related to the figure, fusing both together using a rule-based algorithm.

Xu et al. [191] tackles a similar task, but for tables instead of figures. They use a BM25-based retrieval model to find sentences related to the text of the table to augment the table input in a concentrative method. They feed the resultant text sequence to pretrained language models to output a caption, or extend a partially generated one in an autoregressive manner.

*Multimodal Summarization.* Unlike the captioning task, multimodal summarization must incorporate multimodal sources, such as image and video as input [175] alongside the text, so is a true multimodal application.

Accounting for tables and figures contained in a scholarly document improves the quality of textual summaries [175]. As figures and tables highlight important messages of a paper, end-to-end multimodal summarization focuses on critical aspects during text summary generation. For example, Li et al. [108] and Chen and Zhuge [27] design a modality-based attention mechanism to summarize a sentence–image pair by attending to different part of the images and text units simultaneously. Similarly, the multimodal selective gate network proposed by Li et al. [109] considers both textual input as well as visual features at the global, grid, and object levels.

Accounting for multimodal input also enables multimodal summary output, i.e., summaries containing both text and non-text modalities. A multimodal summary containing both text and figures provides the user with additional visual information and ease the comprehension process as compared to a text-only summary [213]. In addition to text, summarization incorporating multimodal output system includes an image selector to include images into the summary based on evidence from both the input text and images. Zhu et al. [213] propose a multimodal attention model to jointly generate summary text and select the image that has the highest visual coverage [108]. Chen and Zhuge [27, 28] follow a similar framework in their two works, also considering both the visual features and the ordering of those components in generating visual representations. They accomplish this by encoding the visual features with a CNN model, followed by an RNN model for the ordering representation. To further improve the multimodal representation, Zhang et al. [211] proposed a unified approach to handle multimodal input by incorporating both multimodality and knowledge distillation in representation.

Yet with these advances, there is still much room for improvement. Performance of multimodal summarizers degrade in the face of two connected problems: modality bias and insufficient data (resources). The problem of modality bias occurs when models are optimized to generate good text summaries but ignore the quality of the selected images during training [215]. Zhu et al. [215] design a multimodal objective function to include the image selection loss in the training objective. Ye et al. [204] further show that adding a residual connection to the model effectively alleviates modality bias. Methods to improve the performance in low-resource settings include utilizing cross-domain dataset and incorporating unsupervised training. One way to incorporate pretrained models proposed by Yamamoto et al. [195] is to build visual-based image selector and language-based text generator as two individual modules. While this setup allows both modules to be trained with other datasets, the improvement brought by fusing the other modality in each task is neglected. To address this, Zhu et al. [214] project the embeddings of the textual and visual information to a common semantic space and estimate the text–image similarity using a pretrained captioning model. They then build a graph-based extractive summarizer where the similarity (as edges) between different information units (as nodes) is unsupervisedly trained.

Summaries can also take special forms in scholarly documents: as posters and presentation slides, which are themselves multimodal views of a scholarly document. A poster contains several panels, each covering a section of the source document and containing both text and figures. A poster generator needs to identify the important sections and extract the salient content for the panel. The poster generation model by Xu and Wan [192, 193] learns to predict important text and figures simultaneously based on their section-aware representations. The extracted panels are then used to fill the predefined template to form the poster. Similarly, a presentation slide deck can inherit the structure of the source document. It involves multimodal summary extraction and paraphrasing of the source content to be more concise [55]. Furthermore, the identified information units need to be arranged into the layout of presentation slides. The system proposed by Fu et al. [55] contains modularized components for each of these subtasks, and trains the model end-to-end using a multiobjective loss function covering both content selection and layout. In contrast, Sun et al. [168] features a human-in-the-loop, letting users input slide titles to retrieve the most relevant sentences and figures to those titles. They use a question answering paradigm, where the slide titles function as questions and retrieve sentences as the source passage for paraphrasing into concise forms.

*Recommendations*: In reviewing the prior work in multimodal SDP, we notice an alignment gap. The practical work in multimodal SDP applications we reviewed here (Sect. 5.2) have largely neglected the importance of appropriate multimodal representation (Sect. 5.1). This suggests a significant pathway forward: where the performance of multimodal applications—such as our reviewed ones of captioning and summarization—improve by careful and appropriate choice of their multimodal representation. We note that in work related to both applications, there has also been a focus on appropriate selection: body text is long and laden with discourse signals that help localize relevant text. Summarization is an application also featured in Challenges #1 and #2, such that both discourse structure and citation networks can be thought of as modalities themselves. This suggests that fusing representations across all three challenges may benefit summarization performance.

In the larger context, we believe that the scope of what multimodality is defined as in current scientific document processing is still limiting. As SDP matures, a broadened scope that includes the entire spectrum of modalities—*inlined, internal* and *external* artifacts—will afford new and interesting artifact-centric (i.e., equation, dataset, and grant funding mention indexing) functionalities.

## Resources

The proliferation of neural methods in SDP has spurred the creation of many new large-scale datasets, and tools to train and deploy modern neural network models and make them accessible to downstream practitioners.

**Table 3:** SDP-related tools and frameworks, characterized based on (a) **SDP tasks**: which tasks are directly supported, (b) **Neural support**: whether they include neural network-based models, (c) **PDF processing**: whether they support processing PDF pipelines, (d) **Experimentation**: whether they allow researchers to experiment with the machine learning models, (e) **End-user application**: whether they provide mechanisms to deploy models, and (f) **Code Availability**: The availability of the project code

Tool	SDP tasks	Neural support	PDF processing	Experimentation	End-user application	Code availability
CERMINE [174]	Extract reference string, Citation string parsing		$\checkmark$			$\checkmark$
GROBID [116]	Header parsing, Reference extraction and parsing, Citation context recognition, Citation string parsing, Logical structure recovery		$\checkmark$			
ParsCit [37]	Logical structure recovery, Header parsing, Citation string parsing		$\checkmark$		$\checkmark$	$\checkmark$
Neural-ParsCit [148]	Citation string parsing	$\checkmark$				$\checkmark$
Scienceparse$^{4}$	Logical structure recovery	$\checkmark$	$\checkmark$			$\checkmark$
SciSpacy [140]	Biomedical named entity recognition, Biomedical named entity linking				$\checkmark$	$\checkmark$
SciWING [153]	Logical structure recovery, Header parsing, Citation string parsing, Citation intent classification, Clinical notes parsing	$\checkmark$	$\checkmark$	$\checkmark$	$\checkmark$	$\checkmark$

The url to the code is listed in the reference for those available https://github.com/allenai/spv2

### Tools and frameworks

Many recent generic NLP tools, like AllenNLP [59], FLAIR [5], Texar [81], Spacy^4^ have been built on top of deep learning frameworks like TensorFlow [1] and Pytorch [145]. However, they do not cater to the specific needs of SDP. We consider tools and frameworks specific to SDP and use the following dimensions to compare them as shown in the columns of Table 3: applicable SDP tasks, neural support, PDF support, experimentation support, and end-user applications. We make the following observations:

1. *SDP tasks*: To be useful in the real world, we need to combine solutions from different SDP tasks. An ideal package for practitioners would provision coverage for a large number of SDP tasks. From the survey, we find that GROBID and SciWING [116, 153] lead on this front, while others like Neural-ParsCit [148] and Science Parse deal with specific tasks like citation string parsing and logical structure recovery. Future tools and frameworks should be engineered to facilitate the easy addition of SDP tasks to provide better coverage.
2. *Neural support*: There still is a significant gap in making recent neural methodologies easy to apply for downstream domains such as SDP. We call for the community to continue to add to or support frameworks to help researchers and practitioners obtain the fruits of these benefits. Some teams have chosen to reinvent their frameworks anew. Neural-ParsCit [148], Science Parse and SciWING [153] provide native access to neural network pretrained models for end-users. Others such as GROBID have taken the path of retrofitting neural network methods into their frameworks. GROBID [116] also provides end-users with neural network-based methods.
3. *PDF processing*: Most of the scientific documents are available in the Portable Document Format (PDF). Tools that provide end-to-end PDF processing pipelines improves ease of use. Otherwise, preprocessing to obtain the needed input representation from the PDF is first required. Given the integral nature of processing PDFs for SDP tasks—for example, to identify discourse structure—tools like CERMINE [174], GROBID [116], ParsCit [37], and SciWING [153] include mechanisms to directly ingest PDFs. However, most of these tools treat PDFs as text-only documents and do not consider the computer vision and multimodal methods that have shown improved performance in other application domains. This current weakness is a key area we feel for improvement that the community should prioritize.
4. *Experimentation*: Many neural network models are a precise combination of different modules and embeddings; even applications to related SDP tasks and domains often exhibit lower performance due to the need for extensive tuning. Both SDP researchers and practitioners would benefit from allowing experimentation of the model, to tune the embeddings, and to train and fine-tune the models on their own application domain’s data. Except for SciWING [153], most tools in our list and frameworks do not allow for such experimentation.
5. *End-user application*: Most SDP practitioners are interested in obtaining results for downstream use, treating the software as off-the-shelf solutions with easy to use, and may not be concerned in tuning the models. Providing end-user applications are essential to achieve this. Most of the tools like Science Parse and Neural-ParsCit [148] are the result of research efforts, so do not focus on providing end-user applications. Even trivial interfaces provided by ParsCit [37], SciSpacy [140] and SciWING [153] are useful. Further, tools like scite.ai^5^ and Semantic Scholar that integrate the end results of research into a user-friendly website is already proving beneficial for scholar practitioners.

### Datasets

We also summarize the recent efforts to provide large-scale datasets addressing challenges in SDP. Again, while there are limitless possible SDP tasks, it is instructive to limit our discussion to tasks (and their associated resources) centrally related to our three challenges. For these reasons, in this survey, we examine SDP datasets targeting the two tasks of **summarization** and **citation intent classification**.

**Table 4:** Large-scale SDP summarization datasets

Dataset name	Long/short	Size (# summaries)	Single/multiple	Abstractive/extractive	Dataset availability
SciTLDR [16]	Short	1700	Single	Abstractive	$\checkmark$
SciSummNet [202]	Long	1K	Single	Abstractive	$\checkmark$
BIGPATENT [159]	Long	1 M	Single	Abstractive	$\checkmark$
TALKSUMM [106]	Long	1.7K	Single	Extractive	$\checkmark$
CSPubSum [35]	Long	10K	Single	Extractive	$\checkmark$
MS$^2$ [45]	Long	470K	Multi	Abstractive	$\checkmark$
arXiv [33]	Long	215K	Single	Abstractive	$\checkmark$
PubMed [33]	Long	133K	Single	Abstractive	$\checkmark$
LaySumm [20]	Long	572	Single	Abstractive	$\checkmark$
LongSumm [20]	Long	2.2K	Single	Both	$\checkmark$

We compare them based on: (a) **Long**/**Short**: we consider any summary greater than a mean length of 50 words a *long* summary, (b) **Size**: The number of document summary pairs, (c) **Single**/**Multiple**, (d) **Abstractive**/**Extractive**: Whether the summaries are extractive or abstractive. **Dataset availability** refers to availability of the paper’s dataset; where available, URLs are listed in the respective reference in the bibliography

#### Summarization

Progress in practical summarization has taken large strides in the past few years, spurred by the aforementioned techniques to train large-scale models and the availability of data [44, 156]. Neural network approaches have particularly targeted abstractive forms of summarization, but require large amounts of data. For news article summarization, corpora such as CNN/Daily Mail [76] and NewsRoom [65] are examples. However, providing large-scale human-annotated summaries datasets for scientific documents is expensive. They are long documents and require human subjects to have a complete understanding of the document before creating a good summary. In recent years, the SDP community has invested efforts to create larger datasets [20, 21], for which we provide an overview here:

1. *SciTLDR* [16]: introduces a TLDR (“Too long; didn’t read”) dataset for 1–2 sentence summaries of scientific publications, suitable for presenting as search snippets. They obtain TLDRs from peer reviews culled from OpenReview^6^—a platform for authors and independent reviewers. Such extreme forms of summarization for scientific publications are reminiscent of their news article counterparts in XSUM [138].
2. *SciSummNet* [202]: creates a human-annotated scientific summarization dataset by asking the annotators to read the abstract of a paper and all the citing sentences and form a summary. This dataset contains 1,000 paper–summary pairs.
3. *BIGPATENT* [159]: Like scientific documents, patents are long-form, conventionally structured documents. BIGPATENT uses the abstract of a patent as its summary. It includes more than a million document–summary pairs.
4. *TALKSUMM* [106]: considers the video recordings of paper presentations in NLP and machine learning-based conferences. They align the sentences in the video transcripts with sentences from the paper using Hidden Markov Models and use these sentences as extractive summaries. Models trained on the automatically extracted dataset are as performant as on human-annotated data. The dataset contains 1700 paper–summary pairs.
5. *CSPubSum* [35]: use the highlight statements provided by authors of ScienceDirect^7^ publications as human-annotated extractive summaries. Further, they also extend the gold summary sentences, by considering the top sentences that have a high ROUGE-L [114] scores. The dataset consists of summaries for more than 10,000 papers.
6. *MS*$^2$ [45]: Medical studies are also a form of scientific documents that have started to garner attention. The Multi-Document Summarization of Medical Studies (MS$^2$) dataset features medical articles and summaries to investigate the summarization and sensemaking of possibly contradictory biomedical articles. It also provides annotation of key clinical medical metadata in the form of patient, intervention, comparison, and outcome (PICO [82]) keyphrases. Such datasets represent the recent wave toward multitask and joint learning, where two SDP tasks can profitably benefit each other.
7. *ArXiv and PubMed datasets* [33]: introduces the ArXiv and the PubMed datasets for summarization. They consider the abstracts as the summaries and the entire scientific article as the source. Since the abstracts are written by humans, the summaries are considered abstractive. The ArXiv dataset contains more than 200,000 articles while the PubMed dataset—contains more than 133,000 articles, making these datasets some of the largest available.
8. *LongSumm* [20]: Most of the summarization datasets include summaries that are a few hundred words. A longer summary that enables one to explore the research article—such as research weblogs—are lacking. LongSumm aims to tackle this challenge by contributing 1705 extractive summaries from the previous [106] dataset, also accompanied by abstractive summaries originating from research blogs that contain on average of 30 or more sentences. It formed one of the SDP shared tasks in 2021, chalking up 18 submissions to its three tasks.
9. *LaySumm* [20] To make science more accessible to non-technical readers, LaySumm aims to produce summaries that explain the overarching goal and impact of a scientific document. This dataset contains around 570 human-written lay summaries of scientific documents and the corresponding abstract and full text are made available.

**Table 5:** Datasets for citation intent classification

Dataset name	Label space	Size	Labeled citation context	Dataset availability
Abu-Jbara et al. [3]	Criticizing, comparison, using, substantiating, bias, other	3.5K	$\checkmark$	
Cohan et al. [32]	Background, method, comparison	11K		$\checkmark$
Jurgens et al. [87]	Background, motivation, using, extending, compare and contrast, future	1.9K		$\checkmark$
Lauscher et al. [103]	Background, motivation, using, extending, similarities, difference, future	12.6K	$\checkmark$	$\checkmark$
Nambanoor Kunnath et al. [136]	Background, motivation, using, extending, compare and contrast, future	4K		$\checkmark$
Su et al. [164]	Weakness, comparison and contrast, positive, negative	1.4K		$\checkmark$
Valenzuela et al. [176]	Related work, comparison, using, extending	465		

We compare them based on (a) **Label Space**: The set of labels used to classify the citation, (b) **Size**: The number of citations annotated with the citation intent, (c) **Labeled Citation Context**: Indicates whether the dataset also provides annotation for the context of the citation. **Dataset Availability**: Where the dataset is publically available, its hyperlink is listed in its bibliographic reference

Table 4 compares the summarization datasets among a few salient dimensions. (1) ***Long/Short***: The SDP community aims to provide long summaries compared to other domains such as news articles. This is because scientific articles need to include multiple facets to facilitate reasonable comprehension. For example, the summary should help readers understand the context, the problems and gaps in the literature, and the scope of the current article in solving it. (2) ***Size***: News article summaries contain millions of document–summary pairs, compared to a few thousand supervised pairs for scientific articles. Large transformer model that are in vogue for summarization [107, 152, 210] require large-scale data for training. Although recent efforts have been directed in curating large-scale datasets for summarization [16], continued efforts will benefit scientific document summarization. (3) ***Single/Multiple***: Most works consider single documents for summarization, and do not consider the citations or the citing article for summarization. With the increasingly large network of scientific articles and accompanying citations, capturing salient information from multiple related documents provides an alternative form of summarization that is unique to scientific documents. Such summaries will place the scientific document in an appropriate context with respect to other works. (4) ***Extractive/Abstractive***: While the recent application of neural models have improved abstractive summarization, key issues for scientific article summarization remain unaddressed. For example, there is no guarantee of the factuality of the generated summaries. We note that recent methods take steps to address this [29, 137] and going forward, a summary’s fidelity will remain an important criterion in evaluating summaries.

#### Citation intent classification

Analyzing the citations made for a scientific publication can help researchers understand how the scientific community perceives a scientific article. Online scientific platforms such as Semantic Scholar and scite.ai$^{6}$ have deployed such analyses to aid researchers. Table 5 details the efforts to curate datasets for such citation analysis. We compare them with respect to their (a) **Label Space**: Intents annotated by the dataset, (b) **Size**, and (c) **Labeled Citation Context**: whether the citation context is also annotated.

1. *Label space*: Datasets use disparate labels, and some feature a hierarchical taxonomy. Building upon previous works, some datasets break a label into a more fine-grained label. For example, Lauscher et al. [103] breaks down compare and contrast further into *Similarities* and *Differences*. Cohan et al. [32] compose many categories defined by Jurgens et al. [87] into the *Background* section. The common reasons cited by authors are ease of use or observations without any evidence. The recent C3 shared tasks also labeled citation influence (importance), appealing to solutions featuring joint predictions of both tasks Nambanoor Kunnath et al. [136]. Unfortunately, many of these works do not build upon others, fragmenting the datasets and making fair comparison difficult. We suggest that the community rally around a common, simple label space, but which can be extended for discipline-specific needs.
2. *Size*: The largest of datasets has close to 13,000 labeled citation contexts. Compared to well-known text classification datasets, this scale is at least a magnitude smaller, insufficient for building high-performing neural models. This highlights the challenge to employ neural models for this task. Techniques that require special treatment to handle lack of data, are yet to be applied for citation intent classification. Provided that annotating large-scale datasets has been difficult up to now, we see an outlook where such problems are addressed not with additional data, but with data-efficient techniques.
3. *Labeled Citation Context*: While it has been repeatedly shown that citation context improves citation intent classification, currently only two datasets also annotate the citation context [3, 103]. Abu-Jbara and Radev [4] propose to identify citation context automatically, which is not tackled by current neural network methods. Apart from curation efforts to label citation context, automatically identifying the context should be part of the pipeline for citation intent classification.

## Conclusion

We have given an overview of the challenges offered by scientific document processing (SDP). In addition to these key challenges, we conclude by discussing the limitations of our survey and our view of future trends and outlook for scholarly document processing.

*Survey scope limitations*: Our view of SDP, as envisioned in this article, is still limited toward work related more closely to the natural language processing (NLP) community: with intrinsic document and citation processing. This is consistent with the vision of a large subset within the digital library community, e.g., [124, 134]. Our intent was to provide a comprehensive viewpoint on this scope; accordingly, our discussion of the tasks, terminology, and datasets is limited to the scenarios mentioned here.

However, SDP can be construed more broadly to account for relevance to any textual processing involved in scholarly documents, including its multimodality—visual and aural [144]), its auxiliary artifacts (that is, data and software [18, 77] or controlled metadata [125, 141])—and its archiving and preservation [54]. A key limitation of our work is that we have purposefully omitted discussion of issues related to these other areas, and leave the generalization to future scholars. Importantly, we believe that the three challenges we have identified are still entirely relevant to all such research and application areas.

*Future outlook*: Are there other key issues and contexts that the SDP community needs to consider in the upcoming years of development, within the scope of the three challenges described here? Emphatically, yes!

To conclude, we offer our point of view on five critical issues that the community should address.

1. *Lack of deep learning tools for SDP*: The proliferation of modern learning methods within the NLP community has had a deep and lasting impact. However, the use of such advances within the SDP community has been difficult [64]. To facilitate sharing the advances of deep learning on SDP tasks, there is a clear need for easy-to-use tools. In Sect. 6.1, we saw that neural network methods are integrated in a few frameworks. But they are siloed, address only a limited number of tasks, and have a steep learning curve. To enable researchers to adopt modern methods in SDP, there is a dire need for tools that provide pretrained models and allow easy experimentation with minimal changes, parallel to general NLP open-source projects.
2. *Minimal supervised data*: Abundant data is one of the reasons for the success of large-scale neural networks. Annotating data to obtain large-scale supervised data is an expensive venture that requires domain expertise, money, and effort. Therefore, researchers continue to work in making neural networks effective in low-data scenarios. Pretraining and fine-tuning domain-specific transformer models is currently the most effective and popular way to make modern neural methods work in new domains [70]. With pretraining becoming ubitiquous in NLP applications, more studies such as Gupta et al. [69] that examine its effects of SDP tasks are needed. Data augmentation is another popular technique to increase the size of the dataset [6, 62, 128, 155]. Alternate learning mechanisms such as active learning [92, 186] that reduces annotation costs and multitask learning that results in more generalized models can also alleviate data scarcity problems [38, 63, 163, 183, 217]. On the other hand, large language models can be used as a tool to alleviate the burden of annotating by producing annotations in a semiautomatic manner [48]. Working with minimal supervised data is an important endeavor for machine learning in general. Solutions developed to this problem should be adopted by SDP, and generally help the scientific community.
3. *Knowledge driven methods*: Although advances in deep learning generative methods produce fluent language, it suffers from hallucinations and other text degeneration problems [179]. Also, it does not ensure that the generated text is factual and that the important terms from the source document are not missed—critical for scientific documents. Summarization has especially seen an influx of work that ensures that the generated summaries are factual (in the non-SDP context) and ensures that important facts are not omitted from the summaries [121]. Another important area where factuality is important is question answering [158] and fake science detection [104]. Knowledge bases, which are mostly manually curated concise representations of real-world knowledge, can help ensure that neural networks outputs are factually correct. Many modern neural network methods inject side information from knowledge bases into their architectures [115]; for example, for summarization systems [66, 212] and for question answering [158]. Integrating knowledge graphs into neural network representations is an interesting recent endeavor, which will continue to gain importance in the future and is especially important for scientific document processing.
4. *Understanding long and multiple documents*: Scientific documents are long and complex documents that pose major challenges to the current neural network architecture. Recent efforts have taken different approaches to improve the number of tokens analyzed and produced by models [14, 207]. Additionally, to assist in understanding a scientific field and automating literature reviews, the SDP community needs to research work that goes beyond single documents. Neural network representations can consider other related documents. In this vein, Cohan et al. [34] introduce the SPECTER model, which uses contrastive learning to learn similar representations for closely related documents. This concept presents multiple challenges to the current neural network paradigm, such as increasing computational time and cost. These tasks serve as an appropriate testbed to understand the advances on these fronts, as with even longer documents, such as work on theses and dissertations [53].
5. *For humans, by humans*: SDP aims to make science faster and better. The importance of SDP has increased with the collaborative work on COVID-19 [93, 131]. The community can use these urgent necessities to motivate work to further streamline common research goals so that researchers can spend more quality research time working on difficult cognitive tasks. One way to achieve this is to help researchers perform literature review [182], write scientific papers efficiently [181], recommend papers [52], produce automated summaries [16], understand the context of a problem, and write critiques of a paper. Progress in SDP is of little use if such human-assistive technologies are not adopted outside research. Digital libraries need to deploy such works to enable researchers to be more efficient. Automation allows efficiency, but the SDP community also needs to engineer suitable work and evaluation processes for check and balances of the quality of automation. Advanced automation poses difficulty for evaluation as tasks become harder to judge, especially with respect to recall (missing key work or insights). Authors of productionized SDP tools have an advantage for understanding and creating insights that further their own research, possibly creating imbalances that unfairly discriminate against junior and non-native researchers. This is a recognized problem in general language technology deployments and is being actively addressed in the NLP community through a series of workshops [10, 39, 177]. We need to address these fundamental of diversity and inclusion issues before they become endemic problems in scientific research.

## Footnotes

1 https://semanticscholar.org.

2 https://www.aminer.org/.

3 https://lilianweng.github.io/lil-log/2020/04/07/the-transformer-family.html.

4 https://www.spacy.io.

5 https://www.scite.ai.

6 https://openreview.net/.

7 www.sciencedirect.com.
